# The Neutrophil-to-Lymphocyte and Monocyte-to-Lymphocyte Ratios Are Independently Associated With the Severity of Autoimmune Encephalitis

**DOI:** 10.3389/fimmu.2022.911779

**Published:** 2022-07-01

**Authors:** Zhiwei Liu, Yimeng Li, Yaoyao Wang, Haifeng Zhang, Yajun Lian, Xuan Cheng

**Affiliations:** Department of Neurology, The First Affiliated Hospital of Zhengzhou University, Zhengzhou, China

**Keywords:** autoimmune encephalitis, neutrophil-to-lymphocyte ratio, monocyte-to-lymphocyte ratio, the Clinical Assesment Scale for Autoimmune Encephalitis, the modified Rankin Scale, severity

## Abstract

**Background:**

The neutrophil-to-lymphocyte ratio (NLR) and monocyte-to-lymphocyte ratio (MLR) are biomarkers that may reflect inflammatory status in some immune-related diseases. This study aims to investigate the association of NLR and MLR with the severity and prognosis of autoimmune encephalitis (AE).

**Methods:**

A total of 199 patients diagnosed with AE in the First Affiliated Hospital of Zhengzhou University from October 2015 to October 2021 were retrospectively analyzed. The Clinical Assessment Scale for Autoimmune Encephalitis (CASE) and the modified Rankin Scale (mRS) were used to evaluate the severity of the patients at admission, and the patients were divided into mild group (CASE ≤ 4) and severe group (CASE ≥ 5) according to the CASE score. Poor prognosis was described as an mRS of 3 or more at 12 months. Binary logistic regression analysis was performed to assess risk factors for the severity and prognosis of AE.

**Results:**

NLR and MLR of severe group were significantly higher than that of mild group. NLR and MLR were positively correlated with the CASE score (*r* = 0.659, *P* < 0.001; *r* = 0.533, *P* < 0.001) and the mRS score (*r* = 0.609, *P* < 0.001;*r* = 0.478, *P* < 0.001) in AE patients. Multivariate logistic analysis showed that NLR (OR = 1.475, 95%CI: 1.211-1.796, *P* < 0.001) and MLR (OR = 15.228, 95%CI: 1.654-140.232, *P* = 0.016) were independent risk factors for the severity of AE. In addition, the CASE score and the mRS score were positively correlated (*r* = 0.849, *P* < 0.001). Multivariate logistic analysis showed that the CASE at admission (OR = 1.133, 95%CI: 1.043-1.229, *P* = 0.003) and age (OR = 1.105, 95%CI: 1.062-1.150, *P* < 0.001) were independent risk factors for the poor prognosis of AE patients. The NLR and MLR at admission and whether they decreased after immunotherapy were not associated with the prognosis of AE patients (*P* > 0.05).

**Conclusions:**

NLR and MLR, readily available and widespread inflammatory markers, were helpful for clinicians to monitor disease progression and identify potentially severe patients of AE early to optimize clinical treatment decisions.

## 1 Introduction

Autoimmune encephalitis (AE) is an autoimmune inflammatory disease targeting neuronal cell surface or synaptic proteins in the central nervous system (1). Dalmau et al. (2) first discovered anti-N-methyl-D-aspartate receptor (NMDAR) antibodies in patients with encephalitis in 2007 and proposed the concept of AE. Subsequently, increasing subtypes of antibody-mediated encephalitis such as leucine-rich glioma inactivated 1 (LGI1) encephalitis and γ-aminobutyric acid type B receptor (GABA_B_R) encephalitis have been discovered. The clinical manifestations of AE are complex and diverse, mainly including seizures, psychiatric and behavior disorders, consciousness disorders, speech disorders, autonomic nervous dysfunction, cognitive dysfunction and involuntary movements (3). Furthermore, AE is a disease that has significant clinical heterogeneity, and some patients progress rapidly, which may be life-threatening due to central hypoventilation or severe autonomic nervous dysfunction within weeks or even days (4, 5). However, studies on biological indicators of the severity and prognosis of AE is still in its infancy, and an objective and inexpensive biomarker is urgently needed to guide clinical practice.

The neutrophil-to-lymphocyte ratio (NLR) is a biomarker derived from blood routine to reflect the inflammatory status of the body. Previous studies on NLR were mostly related to tumors. A Meta-analysis in Canada showed that cancer patients with high levels of NLR tend to have a poor prognosis (6). NLR is gradually found to be closely associated with the severity and poor prognosis of AE patients (7–10). Nevertheless, in these studies, patients were assessed using the modified Rankin Scale (mRS), which takes motor function as the main indicator. In addition to motor dysfunction, AE includes a variety of non-motor symptoms such as psychiatric and behavior disorders, seizures, consciousness disorders and speech disorders. Therefore, there are great limitations in the assessment of AE with the mRS.

The Clinical Assessment Scale for Autoimmune Encephalitis (CASE) is a new assessment scale designed specifically by Lim et al. (11) for AE in 2019, which compensates for the deficiencies of the mRS in assessing non-motor symptoms of AE. Moreover, the criteria of the CASE are more detailed and specific, which can assess whether the severity of disease in different stages changes or not more accurately. The validity of the CASE has been verified in several studies (12, 13).

The monocyte-to-lymphocyte ratio (MLR) is a new inflammatory marker similar to NLR discovered in recent years. A Korean study on endometrial cancer displayed that high levels of MLR are prominently associated with cancer recurrence and cancer-related death (14). Interestingly, it has also been found to be associated with the severity and activity of some immune-related diseases such as multiple sclerosis, systemic lupus erythematosus, axial spondyloarthritis and ulcerative colitis (15–18). However, it is unclear whether MLR is associated with the severity and prognosis of AE. Therefore, this study aims to analyze the clinical characteristics of AE and whether NLR and MLR are associated with the severity and prognosis of AE.

## 2 Subjects and Methods

### 2.1 Patients

This study included 199 patients diagnosed with AE in the First Affiliated Hospital of Zhengzhou University from October 2015 to October 2021. This study was approved by the ethics committee of the First Affiliated Hospital of Zhengzhou University and followed the Declaration of Helsinki. The patients included in the study fulfilled the criteria were as follows: (1) met the diagnostic criteria for AE proposed by Graus et al. (19) in 2016; (2) serum and/or cerebrospinal fluid (CSF) testing positive for antibodies with AE; (3) complete clinical data. The following exclusion criteria were considered: (1) comorbidity with other systemic autoimmune diseases such as systemic lupus erythematosus and Sjogren’s syndrome; (2) comorbidity with diseases that may affect blood routine, such as severe infection and hematological diseases; (3) comorbidity with other diseases resulting in neurological dysfunction; (4) possible AE was diagnosed, but examination of AE related antibodies was not performed.

### 2.2 Data Collection

Data on age, gender, clinical manifestations, immunotherapy, and routine blood tests were collected. Routine blood tests include white blood cell (WBC), neutrophils, monocytes and lymphocytes. NLR = neutrophils/lymphocytes; MLR = monocytes/lymphocytes. The routine blood tests were performed in all patients within 24 hours after admission and before immunotherapy. Patients were followed up and routine blood tests were obtained after immunotherapy. The median duration of follow-up for routine blood tests was 27 days.

### 2.3 Evaluation of Disease Severity at Admission and Prognosis of Patients With AE

The primary endpoints were disease severity (as assessed by the CASE and mRS) at admission and prognosis (as assessed by the mRS at 12 months). The CASE scale is divided into nine items, including seizures, memory dysfunction, psychiatric symptoms, consciousness, language problem, dyskinesia/dystonia, gait instability and ataxia, brainstem dysfunction, and weakness. Brainstem dysfunction included gaze paresis, tube feeding, and ventilator care due to central hypoventilation. The total scores of the CASE are 27 points. All patients were divided into mild group (CASE ≤ 4) and severe group (CASE ≥ 5) according to the CASE score at admission. The scale was independently evaluated by two neurologists who were unaware of the diagnosis through studying the detailed medical records described by the neurologists and nurses, retrospectively. Poor prognosis was described as an mRS of 3 or more at 12 months.

### 2.4 Statistical Analysis

All data were statistically analyzed using SPSS version 26.0 (IBM, Chicago, IL, USA). Normally distributed continuous variables were defined as mean ± standard deviation (SD), and independent samples t-test was used for comparison between two groups. Non-normally distributed continuous variables were defined as median and interquartile range (*M*, IQR) and compared by Mann-Whitney U test between two groups and Kruskal-Wallis H test among three groups. Bonferroni correction was used for multiple comparisons among the three groups. The chi-square test was used to compare categorical variables expressed as numbers or percentages. Spearman correlation analysis was used to test the correlation of NLR and MLR with the disease severity. Receiver operating characteristic (ROC) curve was used to evaluate the power of NLR and MLR in predicting the severity of AE, and the area under the curve (AUC) was calculated. Logistic regression was used to analyze risk factors for the severity and prognosis of AE. The level of significance was defined as *P* < 0.05; The significant of Bonferroni correction pairwise comparison was *P* < 0.017.

## 3 Results

### 3.1 Baseline Characteristics of Patients

A total of 199 patients with AE were enrolled in this study, including NMDAR encephalitis (59.8%), LGI1 encephalitis (20.6%), and GABA_B_R encephalitis (19.6%). The age distribution and clinical manifestations of different subtypes of AE are remarkably heterogeneous (**Table 1**). NMDAR encephalitis was more prevalent in younger patients than in other subtypes (both *P* < 0.017). In this study, we observed that the proportion of seizures in NMDAR encephalitis (42.0%) was significantly lower than that in GABA_B_R encephalitis (71.8%)(*P <* 0.017). Consciousness disorders was more common in GABA_B_R encephalitis (61.5%) compared with other subtypes (both *P* < 0.017). The proportion of psychiatric and behavior disorders (46.3%) and speech dysfunction (24.4%) were lower in LGI1 encephalitis compared with NMDAR encephalitis and GABA_B_R encephalitis (both *P* < 0.017). The CASE score and mRS score of patients with LGI1 encephalitis were lower than that of patients with NMDAR encephalitis and GABA_B_R encephalitis, with statistically significant differences in mRS score (*P* < 0.017). The proportion of severe patients with LGI1 encephalitis was significantly lower than that with NMDAR encephalitis (*P* < 0.017). There were no significant difference in gender, WBC, neutrophils, lymphocytes, monocytes, NLR and MLR among this three subtypes (*P* > 0.05).

**Table 1:** Clinical features and laboratory parameters of patients with AE.

Variables	AE (n=199)	NMDAR (n=119)	LGI1 (n=41)	GABA_B_R (n=39)	*P*_1_	*P*_2_	*P*_3_	*P*_4_
Male, n (%)	122 (61.3%)	66 (55.5%)	30 (73.2%)	26 (66.7%)	0.099			
Age (*M*, IQR)	41.0 (24.0-59.0)	28.0 (20.0-41.0)	61.0 (49.5-66.5)	61.5 (54.0-66.3)	**<0.001**	<0.001*	<0.001*	0.946
Seizures, n (%)	102 (51.3%)	50 (42.0%)	24 (58.5%)	28 (71.8%)	**0.003**	0.067	0.001*	0.214
Psychiatric and behavior disorders, n (%)	138 (69.3%)	86 (72.3%)	19 (46.3%)	33 (84.6%)	**0.001**	0.003*	0.121	<0.001*
Consciousness disorders, n (%)	76 (38.4%)	43 (36.4%)	9 (22.0%)	24 (61.5%)	**0.001**	0.088	0.006*	<0.001*
Speech dysfunction, n (%)	104 (52.3%)	74 (62.2%)	10 (24.4%)	20 (51.3%)	**<0.001**	<0.001*	0.229	0.013*
CASE (*M*, IQR)	5 (2-10)	6 (2-13)	3 (2-5)	5 (3-7)	**0.03**	0.018	0.343	0.022
MRS (*M*, IQR)	3 (2-4)	3 (2-4)	2 (2-3)	3 (2-3)	**0.002**	0.001*	0.128	0.015*
Severe patients, n (%)	102 (51.3%)	67 (56.3%)	13 (31.7%)	22 (56.4%)	**0.019**	0.007*	0.991	0.026
WBC (10^9^/L, *M*, IQR)	8.10 (6.40-9.73)	8.15 (6.60-9.95)	8.00 (6.08-9.35)	8.10 (6.02-10.10)	0.734			
Neutrophils (10^9^/L, *M*, IQR)	5.90 (3.91-7.80)	6.05 (3.92-8.19)	5.78 (3.87-7.10)	5.30 (4.07-7.15)	0.643			
Lymphocytes (10^9^/L, *M*, IQR)	1.44 (1.09-1.87)	1.47 (1.10-1.94)	1.38 (1.07-1.67)	1.51 (1.02-1.88)	0.535			
Monocytes (10^9^/L, *M*, IQR)	0.57 (0.43-0.74)	0.57 (0.43-0.72)	0.59 (0.44-0.85)	0.53 (0.40-0.80)	0.664			
NLR (*M*, IQR)	4.20 (2.49-6.10)	4.54 (2.60-6.46)	4.33 (2.40-5.90)	3.59 (2.37-5.09)	0.353			
MLR (*M*, IQR)	0.40 (0.28-0.59)	0.42 (0.28-0.58)	0.38 (0.28-0.69)	0.36 (0.28-0.59)	0.868			

M, median; IQR, interquartile range; CASE, The Clinical Assessment Scale for Autoimmune Encephalitis; MRS, modified Rankin Scale; WBC, white blood cell; NLR, neutrophil-to-lymphocyte ratio; MLR, monocyte-to-lymphocyte ratio; AE, autoimmune encephalitis; NMDAR, anti-N-methyl-D-aspartate receptor; LGI-1, leucine-rich glioma inactivated 1; GABA_B_R, γ-aminobutyric acid type B receptor; P_1_, Compared among NMDAR, LGI1 and GABA_B_R; P_2_, NMDAR vs LGI1; P_3_, NMDAR vs GABA_B_R; P_4_, LGI1 vs GABA_B_R; Significant values (P < 0.05) are highlighted in bold; *, Bonferroni correction pairwise comparison was statistically significant (P < 0.017).

### 3.2 NLR and MLR Were Associated With the Severity of AE

#### 3.2.1 Comparisons of Clinical Data Between the Mild Group and Severe Group

Among the 199 patients, 97 patients (48.7%) were in the mild group and 102 patients (51.3%) were in the severe group according to the CASE score. The WBC, neutrophils, monocytes, NLR and MLR in severe group were significantly higher than that in mild group, and lymphocyte was lower than that in mild group (*P* < 0.05). There was no statistical difference in age, gender, and time from first symptoms of disease to hospitalization between the two groups. Further analysis showed that in NMDAR encephalitis, WBC and neutrophils in severe group were higher than that in mild group, lymphocytes were lower than that in mild group (*P* < 0.05), and there were no statistical difference in age, gender, time from first symptoms of disease to hospitalization, and monocytes between the two groups; for LGI1 encephalitis, age, WBC, neutrophils and monocytes in severe group were higher than that in mild group (*P* < 0.05), and there were no significant difference in gender, time from first symptoms of disease to hospitalization, and lymphocytes between the two groups; among GABA_B_R encephalitis, lymphocytes in severe group were lower than that in mild group (*P* < 0.05), and there were no statistical difference in age, gender, time from first symptoms of disease to hospitalization, WBC, neutrophils and monocytes between the two groups. Among this three subtypes, the NLR and MLR of severe group were significantly higher than that of mild group (**Table 2**).

**Table 2:** Comparisons of clinical data between mild and severe patients.

Variables		Mild (CASE ≤ 4)	Severe (CASE ≥ 5)	*P*
Age (*M*, IQR)	AE	40.0 (24.0-59.0)	43.0 (22.8-60.3)	0.896
	NMDAR	31.0 (21.0-41.0)	27.0 (20.0-41.3)	0.682
	LGI1	59.0 (47.0-65.0)	64.0 (59.0-67.5)	**0.028**
	GABA_B_R	61.5 (36.3-65.5)	61.0 (54.5-66.0)	0.178
Male, (%)	AE	62.9%	59.8%	0.655
	NMDAR	61.5%	50.7%	0.24
	LGI1	64.3%	92.3%	0.132
	GABA_B_R	64.3%	68.2%	0.819
Time from first symptoms of disease to hospitalization (days, *M*, IQR)	AE	20.0 (7.0-35.3)	18.0 (10.0-32.5)	0.908
	NMDAR	19.0 (7.0-30.0)	16.0 (10.0-28.0)	0.912
	LGI1	15.0 (3.9-30.0)	30.0 (14.0-40.0)	0.064
	GABA_B_R	12.0 (4.0-25.0)	15.0 (6.5-25.0)	0.552
WBC (10^9^/L, *M*, IQR)	AE	7.61 (5.83-9.10)	8.38 (6.70-11.11)	**0.005**
	NMDAR	7.45 (5.73-8.91)	8.51 (6.80-12.24)	**0.003**
	LGI1	7.27 (5.78-9.01)	8.37 (7.65-12.20)	**0.024**
	GABA_B_R	8.52 (6.83-10.38)	7.90 (5.60-9.53)	0.244
Neutrophils (10^9^/L, *M*, IQR)	AE	5.00 (3.64-6.72)	6.22 (4.41-9.39)	**<0.001**
	NMDAR	4.94 (3.31-6.46)	6.60 (4.57-10.31)	**<0.001**
	LGI1	4.87 (3.81-6.79)	6.66 (5.54-10.14)	**0.013**
	GABA_B_R	5.46 (4.22-7.90)	5.29 (3.42-7.08)	0.690
Lymphocytes (10^9^/L, *M*, IQR)	AE	1.57 (1.29-2.08)	1.25 (0.94-1.71)	**<0.001**
	NMDAR	1.60 (1.31-2.10)	1.31 (1.01-1.86)	**0.004**
	LGI1	1.41 (1.15-1.79)	1.20 (0.98-1.50)	0.101
	GABA_B_R	1.77 (1.44-2.58)	1.22 (0.74-1.58)	**0.03**
Monocytes (10^9^/L, *M*, IQR)	AE	0.54 (0.42-0.68)	0.60 (0.45-0.81)	**0.022**
	NMDAR	0.55 (0.43-0.66)	0.60 (0.45-0.74)	0.185
	LGI1	0.54 (0.41-0.77)	0.83 (0.52-1.04)	**0.023**
	GABA_B_R	0.50 (0.37-0.66)	0.51 (0.44-0.82)	0.225
NLR (*M*, IQR)	AE	2.63 (1.96-4.20)	5.26 (3.97-8.71)	**<0.001**
	NMDAR	2.61 (1.93-3.91)	5.40 (4.50-9.95)	**<0.001**
	LGI1	3.17 (2.00-4.60)	5.80 (4.40-9.53)	**0.001**
	GABA_B_R	2.51 (2.14-3.85)	4.18 (3.02-7.70)	**0.008**
MLR (*M*, IQR)	AE	0.31(0.24-0.41)	0.52(0.40-0.70)	**<0.001**
	NMDAR	0.31(0.24-0.42)	0.51(0.40-0.66)	**<0.001**
	LGI1	0.32(0.25-0.50)	0.68(0.48-1.03)	**0.002**
	GABA_B_R	0.28(0.20-0.33)	0.49(0.35-0.68)	**<0.001**

M, median; IQR, interquartile range; WBC, white blood cell; NLR, neutrophil-to-lymphocyte ratio; MLR, monocyte-to-lymphocyte ratio; CASE, The Clinical Assessment Scale for Autoimmune Encephalitis; AE, autoimmune encephalitis; NMDAR, anti-N-methyl-D-aspartate receptor; LGI-1, leucine-rich glioma inactivated 1; GABA_B_R, γ-aminobutyric acid type B receptor; Significant values (P < 0.05) are highlighted in bold.

#### 3.2.2 Correlations of NLR and MLR With Disease Severity of AE

**Figure 1:**
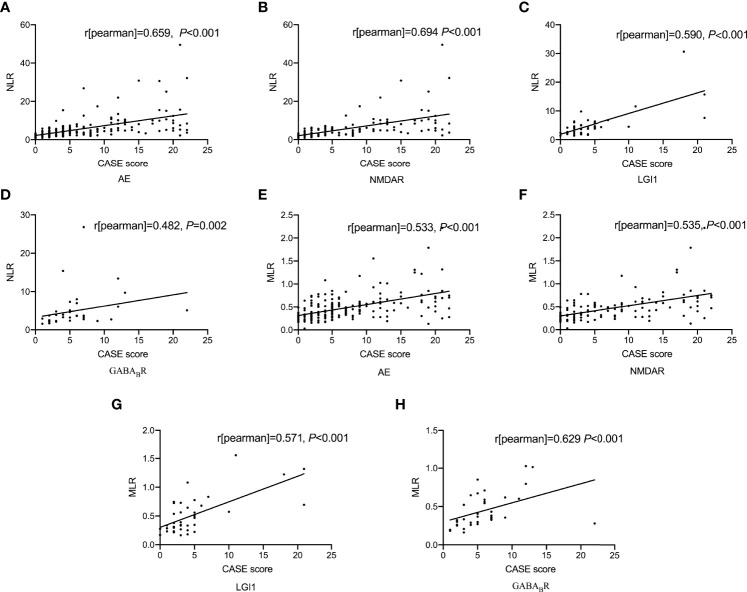
Correlations of NLR and MLR with the CASE score. Correlation between NLR and the CASE score **(A-D)**; Correlation between MLR and the CASE score **(E-H)**. AE, autoimmune encephalitis; NMDAR, N-methyl-D-aspartate receptor; LGI1, leucine-rich glioma inactivated 1; GABA_B_R, γ-aminobutyric acid type B receptor; CASE, The Clinical Assessment Scale for Autoimmune Encephalitis; NLR, neutrophil-to-lymphocyte ratio; MLR, monocyte-to-lymphocyte ratio.

**Figure 2:**
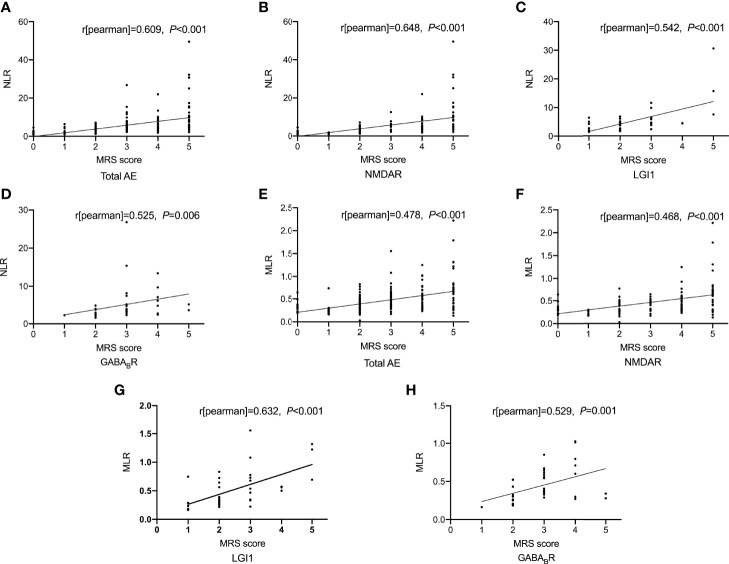
Correlations of NLR and MLR with the mRS score. Correlation between NLR and the mRS score **(A-D)**; Correlation between MLR and the mRS score **(E-H)**. AE, autoimmune encephalitis; NMDAR, N-methyl-D-aspartate receptor; LGI1, leucine-rich glioma inactivated 1; GABA_B_R, γ-aminobutyric acid type B receptor; mRS, modified Rankin Scale; NLR, neutrophil-to-lymphocyte ratio; MLR, monocyte-to-lymphocyte ratio.

**Figure 3:**
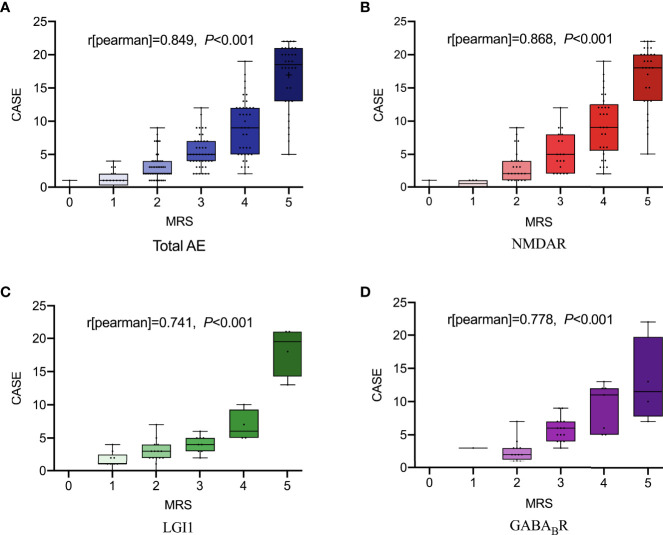
The total scores of the CASE according to the mRS. Total AE, NMDAR, LGI1 and GABA_B_R **(A-D)**. AE, autoimmune encephalitis; NMDAR, N-methyl-D-aspartate receptor; LGI1, leucine-rich glioma inactivated 1; GABA_B_R, γ-aminobutyric acid type B receptor; CASE, The Clinical Assessment Scale for Autoimmune Encephalitis; mRS, modified Rankin Scale.

As shown in **Figure 1**, spearman correlation analysis showed that NLR and MLR were positively correlated with the CASE score in AE patients (*r* = 0.659, *P* < 0.001; *r* = 0.533, *P* < 0.001), and subtype analysis showed that NLR and MLR were positively correlated with the CASE score in NMDAR encephalitis (*r* = 0.694, *P* < 0.001; *r* = 0.535, *P* < 0.001), LGI1 encephalitis (*r* = 0.590, *P* < 0.001; *r* = 0.571, *P* < 0.001), and GABA_B_R encephalitis (*r* = 0.482, *P* = 0.002; *r* = 0.629, *P* < 0.001). We also found that NLR and MLR were positively correlated with the mRS score in AE patients (*r* = 0.609, *P* < 0.001;*r* = 0.478, *P* < 0.001), and subtype analysis showed that NLR and MLR were positively correlated with the mRS score in NMDAR encephalitis (*r* = 0.648, *P* < 0.001;*r* = 0.468, *P* < 0.001), LGI1 encephalitis (*r* = 0.542, *P* < 0.001;*r* = 0.632, *P* < 0.001), and GABA_B_R encephalitis (*r* = 0.525, *P* = 0.006;*r* = 0.529, *P* = 0.001)(**Figure 2**). The CASE scale was validated (**Figure 3**), and the results showed that the CASE score was positively correlated with the mRS score in total AE (*r* = 0.849, *P* < 0.001), NMDAR encephalitis (*r* = 0.868, *P* < 0.001), LGI1 encephalitis (*r* = 0.741, *P* < 0.001) and GABA_B_R encephalitis (*r* = 0.778, *P* < 0.001).

#### 3.2.3 Receiver Operating Characteristic Curve Analysis of NLR and MLR to Evaluate the Severity of AE

**Figure 4:**
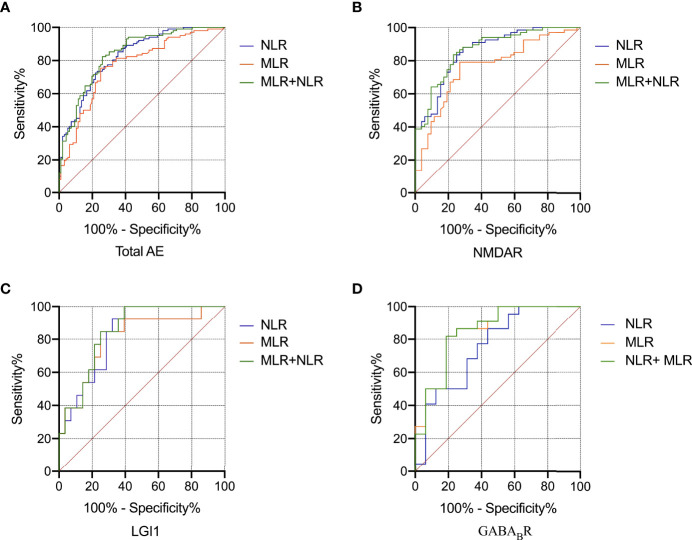
ROC curve analysis of the predictive value of NLR and MLR for the severity of AE. Total AE, NMDAR, LGI1 and GABA_B_R **(A-D)**. AE, autoimmune encephalitis; NMDAR, N-methyl-D-aspartate receptor; LGI1, leucine-rich glioma inactivated 1; GABA_B_R, γ-aminobutyric acid type B receptor; ROC, receiver operating characteristic; NLR, neutrophil-to-lymphocyte ratio; MLR, monocyte-to-lymphocyte ratio.

ROC curve was used to analyze the ability of NLR and MLR to indicate the severity of AE (**Figure 4**). Based on the ROC curve, the optimal cutoff value of NLR as an indicator for predicting severe disease of AE was projected to be 4.29, with a sensitivity of 73.5%, specificity of 77.1%, and AUC at 0.827 (95%CI: 0.771-0.883, *P* < 0.001); the optimal cutoff value of MLR was projected to be 0.40, with a sensitivity of 75.5%, specificity of 75.0%, and AUC at 0.771 (95%CI: 0.706-0.837, *P* < 0.001); An evaluation of the diagnostic value of NLR combined with MLR (NLR +  MLR) gave an AUC of 0.840 (95%CI: 0.786-0.895, *P* < 0.001). Further analysis of the three subtypes revealed that the AUCs of NLR at NMDAR, LGI1, and GABA_B_R encephalitis were 0.859, 0.835, and 0.756; the AUCs of MLR at NMDAR, LGI1, and GABA_B_R encephalitis were 0.763, 0.808, and 0.849; the AUCs of NLR combined with MLR (NLR + MLR) at NMDAR, LGI1, and GABA_B_R encephalitis were 0.866, 0.849, and 0.849 (**Table 3**).

**Table 3:** Receiver operating characteristic curve of NLR and MLR for the severity of AE.

	Variables	AUC	95%CI	Cutoff	Sensitivity	Specificity	*P*
AE	NLR	0.827	0.771-0.883	4.29	73.5%	77.1%	**<0.001**
	MLR	0.771	0.706-0.837	0.40	75.5%	75.0%	**<0.001**
	NLR+MLR	0.840	0.786-0.895	–	–	–	**<0.001**
NMDAR	NLR	0.859	0.793-0.925	3.51	88.1%	71.2%	**<0.001**
	MLR	0.763	0.677-0.850	0.40	79.1%	73.1%	**<0.001**
	NLR+MLR	0.866	0.802-0.930	–	–	–	**<0.001**
LGI1	NLR	0.835	0.715-0.955	4.29	92.3%	67.9%	**0.001**
	MLR	0.808	0.660-0.956	0.43	84.6%	75.0%	**0.002**
	NLR+MLR	0.849	0.734-0.964	–	–	–	**<0.001**
GABA_B_R	NLR	0.756	0.594-0.917	2.62	86.4%	56.2%	**0.008**
	MLR	0.849	0.722-0.977	0.32	86.4%	81.2%	**<0.001**
	NLR+MLR	0.849	0.720-0.979	–	–	–	**<0.001**

AUC, area under the curve; CI, confidence intervals; NLR, neutrophil-to-lymphocyte ratio; MLR, monocyte-to-lymphocyte ratio; AE, autoimmune encephalitis; NMDAR, anti-N-methyl-D-aspartate receptor; LGI-1, leucine-rich glioma inactivated 1; GABA_B_R, γ-aminobutyric acid type B receptor; Significant values (P < 0.05) are highlighted in bold.

#### 3.2.4 Elevated NLR and MLR Were Independent Risk Factors for Disease Severity of AE

Binary logistic regression analysis was used to identify the risk factors of disease severity of AE (**Table 4**). Univariate regression analysis showed WBC, NLR, and MLR correlated with the severity of AE. Variables with significance in univariate regression analysis were included in multivariate regression analysis, and the results showed that NLR (OR = 1.475, 95%CI: 1.211-1.796, *P* < 0.001) and MLR (OR = 15.228, 95%CI: 1.654-140.232, *P* = 0.016) were independent risk factors for disease severity of AE.

**Table 4:** Factors associated with the severity of AE.

	Univariate		Multivariate	
	OR (95%CI)	*P*	OR (95%CI)	*P*
WBC	1.155 (1.052-1.267)	**0.002**	0.958 (0.853-1.076)	0.466
NLR	1.662 (1.390-1.988)	**<0.001**	1.475 (1.211-1.796)	**<0.001**
MLR	146.934 (23.893-903.610)	**<0.001**	15.228 (1.654-140.232)	**0.016**

OR, odds ratio; CI, confidence interval; WBC, white blood cell; NLR, neutrophil-to-lymphocyte ratio; MLR, monocyte-to-lymphocyte ratio; Significant values (P < 0.05) are highlighted in bold.

### 3.3 Logistic Regression Analysis of Factors Associated With Poor prognosis of AE

Some patients were lost to follow-up and had insufficient follow-up (< 12 months), and 156 patients were followed up at 12 months, including 86 patients with NMDAR encephalitis, 32 with LGI1 encephalitis, and 38 with GABA_B_R encephalitis. All patients were divided into good prognosis group (n = 120) and poor prognosis group (n = 36) according to the mRS score at 12 months (**Table 5**). Univariable logistic regression analysis showed that compared with the good prognosis group, the poor prognosis group had higher age and the CASE score at admission (*P* < 0.05). Further analysis showed that in NMDAR and GABA_B_R encephalitis, the age of patients with poor prognosis was significantly higher than that with good prognosis (both *P* < 0.05). The mRS score at admission and neutrophils of patients with poor prognosis in LGI1 encephalitis were significantly higher than that with good prognosis (both *P* < 0.05). The monocytes of patients with poor prognosis was significantly lower than that with good prognosis in NMDAR encephalitis (*P* < 0.05). Among this three subtypes, the CASE score at admission of poor prognosis group were significantly higher than that of good prognosis group. There were no statistical difference in gender, time from first symptoms of disease to hospitalization, first line immunotherapy, second line immunotherapy, WBC, lymphocytes, NLR and MLR between the two groups. Multivariate logistic regression analysis showed that the CASE at admission (OR = 1.133, 95%CI: 1.043-1.229, *P* = 0.003) and age (OR = 1.105, 95%CI: 1.062-1.150, *P* < 0.001) were independent risk factors for the poor prognosis of AE patients. Further analysis showed that age was an independent risk factor for the poor prognosis of patients with NMDAR (OR = 1.084, 95%CI: 1.018-1.155, *P* = 0.012) and GABA_B_R (OR = 1.086, 95%CI: 1.007-1.170, *P* = 0.032) encephalitis (**Table 6**).

**Table 5:** Univariate logistic regression analysis of factors associated with poor prognosis.

Variables		Good prognosis	Poor prognosis	*P*
Age (*M*, IQR)	AE	36 (21-55)	63 (55-68)	**<0.001**
	NMDAR	27 (18-41)	51 (39-61)	**<0.001**
	LGI1	62 (53-66)	68 (57-74)	0.103
	GABA_B_R	55 (37-63)	64 (57-68)	**0.013**
Male, n (%)	AE	74 (61.7%)	24 (66.7%)	0.587
	NMDAR	45 (57.0%)	3 (42.9%)	0.476
	LGI1	19 (73.1%)	5 (83.3%)	0.605
	GABA_B_R	10 (66.7%)	16 (69.6%)	0.851
Time from first symptoms of disease to hospitalization (days, *M*, IQR)	AE	19 (9-34)	20 (10-31)	0.648
	NMDAR	16 (9-30)	21 (10-36)	0.814
	LGI1	30 (13-60)	60 (36-75)	0.212
	GABA_B_R	12 (5-24)	15 (6-25)	0.618
MRS at admission (*M*, IQR)	AE	3.0 (2.0-4.0)	3.0 (3.0-4.0)	0.058
	NMDAR	4.0 (2.0-4.0)	4.0 (2.0-4.0)	0.528
	LGI1	2.0 (2.0-3.0)	5.0 (3.0-5.0)	**0.014**
	GABA_B_R	2.5 (2.0-3.0)	3.0 (3.0-4.0)	0.088
CASE at admission (*M*, IQR)	AE	4.0 (2.0-9.0)	6.5 (4.8-12.3)	**0.011**
	NMDAR	6.0 (2.0-12.0)	13.0 (11.0-20.0)	**0.018**
	LGI1	2.5 (2.0-4.0)	18.0 (5.0-21.0)	**0.03**
	GABA_B_R	3.5 (2.0-5.8)	6.0 (4.0-9.0)	**0.046**
First line immunotherapy, n (%)	AE	108 (90.0%)	29 (80.6%)	0.135
	NMDAR	76 (96.2%)	6 (85.7%)	0.242
	LGI1	20 (76.9%)	5 (83.3%)	0.733
	GABA_B_R	12 (80.0%)	18 (78.3%)	0.898
Second line immunotherapy, n (%)	AE	7 (5.8%)	2 (5.6%)	0.950
	NMDAR	3 (3.8%)	1 (14.3%)	0.242
	LGI1	3 (11.5%)	1 (16.7%)	0.734
	GABA_B_R	1 (6.7%)	0 (0.0%)	–
WBC (10^9^/L, *M*, IQR)	AE	7.70 (6.32-9.09)	8.20 (6.55-11.45)	0.148
	NMDAR	7.70 (6.40-9.30)	7.00 (6.10-14.40)	0.464
	LGI1	7.65 (6.12-8.48)	9.89 (6.87-16.52)	0.061
	GABA_B_R	7.96 (5.92-9.09)	8.20 (6.23-10.28)	0.186
Neutrophils (10^9^/L, *M*, IQR)	AE	5.38 (3.85-6.97)	5.95 (4.41-9.92)	0.901
	NMDAR	5.60 (3.66-7.43)	5.53 (4.12-11.73)	0.917
	LGI1	5.19 (3.91-6.34)	8.27 (5.02-14.69)	**0.040**
	GABA_B_R	5.00 (3.70-6.66)	5.60 (4.17-7.84)	0.362
Lymphocytes (10^9^/L, *M*, IQR)	AE	1.48 (1.20-1.83)	1.25 (0.88-1.78)	0.572
	NMDAR	1.51 (1.18-1.83)	1.09 (0.95-2.45)	0.731
	LGI1	1.41 (1.13-1.66)	1.22 (0.59-1.80)	0.311
	GABA_B_R	1.68 (1.40-2.40)	1.29 (0.88-1.78)	0.864
Monocytes (10^9^/L, *M*, IQR)	AE	0.57 (0.45-0.72)	0.51 (0.40-0.81)	0.137
	NMDAR	0.57 (0.44-0.70)	0.48 (0.38-0.92)	**0.046**
	LGI1	0.59 (0.46-0.82)	0.52 (0.35-1.03)	0.524
	GABA_B_R	0.53 (0.35-0.73)	0.54 (0.40-0.81)	0.763
NLR (*M*, IQR)	AE	3.71 (2.36-5.26)	4.44 (2.67-7.63)	0.118
	NMDAR	4.00 (2.57-5.82)	4.36 (2.42-10.05)	0.777
	LGI1	3.78 (2.38-4.51)	6.93 (4.57-19.42)	0.059
	GABA_B_R	3.02 (2.21-3.96)	3.75 (2.51-6.38)	0.212
MLR (*M*, IQR)	AE	0.36 (0.28-0.53)	0.41 (0.30-0.63)	0.225
	NMDAR	0.41 (0.28-0.53)	0.41 (0.36-0.50)	0.772
	LGI1	0.34 (0.29-0.56)	0.58 (0.23-1.25)	0.295
	GABA_B_R	0.31 (0.20-0.43)	0.38 (0.30-0.63)	0.178

M, median; IQR, interquartile range; CASE, The Clinical Assessment Scale for Autoimmune Encephalitis; MRS, modified Rankin Scale; WBC, white blood cell; NLR, neutrophil-to-lymphocyte ratio; MLR, monocyte-to-lymphocyte ratio; Significant values (P < 0.05) are highlighted in bold.

**Table 6:** Multivariable logistic regression analysis of factors associated with poor prognosis.

	Variables	OR (95%CI)	*P*
AE	Age	1.105 (1.062-1.150)	**<0.001**
	CASE at admission	1.133 (1.043-1.229)	**0.003**
NMDAR	Age	1.084 (1.018-1.155)	**0.012**
	CASE at admission	1.111 (0.967-1.275)	0.137
	Monocytes	4.782 (0.549-41.62)	0.156
LGI1	MRS at admission	0.658 (0.080-5.431)	0.698
	CASE at admission	1.451 (0.789-2.670)	0.232
	Neutrophils	1.079 (0.550-2.115)	0.825
GABA_B_R	Age	1.086 (1.007-1.170)	**0.032**
	CASE at admission	1.218 (0.913-1.624)	0.180

OR, odds ratio; CI, confidence interval; CASE, The Clinical Assessment Scale for Autoimmune Encephalitis; MRS, modified Rankin Scale; AE, autoimmune encephalitis; NMDAR, anti-N-methyl-D-aspartate receptor; LGI-1, leucine-rich glioma inactivated 1; GABA_B_R, γ-aminobutyric acid type B receptor; Significant values (P < 0.05) are highlighted in bold.

### 3.4 NLR and MLR Were Decreased in AE After Immunotherapy

**Figure 5:**
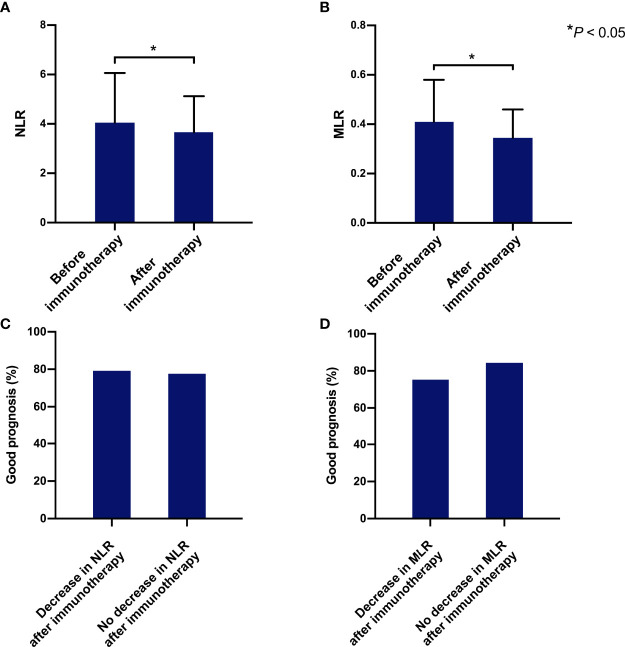
The relationship between the changes of NLR and MLR before and after immunotherapy and the prognosis of patients. The NLR and MLR before and after immunotherapy **(A, B)**; Relationship between whether NLR and MLR decrease before and after immunotherapy and patient prognosis **(C, D)**. * Indicates *P*<0.05.

Using only patients with longitudinal data (n = 126) on NLR and MLR, we tested whether changes in NLR and MLR after immunotherapy could predict the prognosis of patients (**Table 7**). The median duration of follow-up for NLR and MLR was 27 days. The NLR and MLR of patients decrease significantly after immunotherapy (4.05 vs 3.66 and 0.41 vs 0.35, *P* < 0.05)(**Figures 5A, B**). Among the 126 patients, 77 (61.1%) and 81 (64.3%) had decreased NLR and MLR after immunotherapy, respectively. We found that whether NLR and MLR decreased after immunotherapy was not associated with the prognosis of patients (both *P* > 0.05)(**Figures 5C, D**).

**Table 7:** NLR and MLR (before and after immunotherapy).

Variables	Total	Good prognosis	Poor prognosis	*P*
NLR before immunotherapy (*M*, IQR)	4.05 (2.76-6.06)	3.97 (2.81-5.39)	4.69 (2.72-8.85)	0.151
NLR after immunotherapy (*M*, IQR)	3.66 (2.54-5.16)	3.54 (2.41-5.12)	4.34 (3.12-6.13)	**0.033**
NLR D-value (mean ± SD)	1.37 ± 4.97	1.33 ± 4.36	1.53 ± 6.88	0.778
NLR decreased after immunotherapy, n (%)	77 (61.1%)	61 (79.2%)	16 (20.8%)	0.824
MLR before immunotherapy (*M*, IQR)	0.41 (0.29-0.58)	0.41 (0.29-0.55)	0.42 (0.32-0.65)	0.212
MLR after immunotherapy (*M*, IQR)	0.35 (0.27-0.46)	0.34 (0.28-0.46)	0.35 (0.21-0.46)	0.550
MLR D-value (mean ± SD)	0.097 ± 0.249	0.0809 ± 0.233	0.157 ± 0.297	0.358
MLR decreased after immunotherapy, n (%)	81 (64.3%)	61 (75.3%)	20 (24.7%)	0.231

M, median; IQR, interquartile range; SD, standard deviation; NLR, neutrophil-to-lymphocyte ratio; MLR, monocyte-to-lymphocyte ratio; D-value, difference value. Significant values (P < 0.05) are highlighted in bold.

## 4 Discussion

### 4.1 Clinical Features of NMDAR, LGI1, and GABA_B_R Encephalitis

NMDAR encephalitis is the most common subtype of AE, mainly affecting young women. In our study, NMDAR encephalitis was dominated by younger patients, but the sex ratio was almost balanced, which is similar to previous studies in Asian regions such as China and Korea (20, 21). This may be related to ethnicity, economic differences, and the insufficient sample size of this study. The main manifestations of NMDAR encephalitis in this study were psychiatric and behavior disorders, speech dysfunction, seizures and consciousness disorders, which are consistent with previous studies (22). In addition, this study found that the proportion of patients with psychiatric and behavior disorders, speech dysfunction and severe disease, as well as the mRS score of NMDAR encephalitis were significantly higher than that of LGI1 encephalitis, suggesting that the condition of NMDAR encephalitis may be more serious, which is helpful to guide clinicians to formulate appropriate treatment options.

LGI1 encephalitis is the second most common cause of AE after NMDAR encephalitis. It mainly occurs in middle-aged and elderly patients, with a higher proportion of males than females (21, 23). The median age of LGI1 encephalitis in the study was 61 years and mainly affected male patients. Seizures, psychiatric and behavior disorders, consciousness disorders, and speech dysfunction are common symptoms of LGI1 encephalitis, and this study is consistent with previous studies (23). In addition, 19 patients (46.3%) in this study had episodes of Facio-brachial dystonic seizures (FBDS), characterized by unilateral face and/or limb dystonic lasting less than 3 seconds and occurring dozens to hundreds of times per day, which are characteristic of LGI1 encephalitis (24). Therefore, patients with FBDS manifestations found in clinical practice should consider the possibility of LGI1 encephalitis.

Thirty-nine patients with GABA_B_R encephalitis were included in this study, including 26 males (66.7%) and 13 females (33.3%), with a median age of 61.5 years, consistent with previous studies (25, 26). GABA_B_R encephalitis usually presents with seizures as the initial symptom, followed by psychiatric and behavior disorders, consciousness disorders, and memory deficits (27–29). In our study, the proportion of seizures in GABA_B_R encephalitis was significantly higher than that in NMDAR encephalitis. In addition, the proportion of consciousness disorders, psychiatric and behavior disorders, speech dysfunction and the mRS score in GABA_B_R encephalitis were higher than that in LGI1 encephalitis. Compared with LGI1 encephalitis, GABA_B_R encephalitis tended to be more severe, which may be related to the fact that GABA_B_R encephalitis is often associated with tumor, and tumor progression is the leading cause of death in patients with GABA_B_R encephalitis (30). Therefore, patients with GABA_B_R encephalitis should be routinely screened for cancer, and regularly screened for cancer during follow-up, even if no tumor is initially detected.

### 4.2 Elevated NLR and MLR Were Independent Risk Factors for Disease Severity of AE

AE is a highly disabling central nervous system disease characterized by brain parenchymal inflammation and neural circuit damage. Both innate immunity and adaptive immunity play an important role in the occurrence and development of AE (31). The neutrophils and monocytes are representative cells of innate immunity and play an important role in the inflammatory response, but with different mechanisms. Neutrophils are early phase effector cells of autoimmune diseases in the central nervous system, which disrupt the function of blood-brain barrier (BBB) and increase its permeability by releasing a large number of pro-inflammatory factors such as interleukin 1 beta (IL-1β), interleukin 6 (IL-6), tumor necrosis factor alpha (TNF-α) and reactive oxygen species (ROS) (31, 32). In addition, activated neutrophils can induce monocytes recruitment to sites of inflammation by releasing a variety of cytokines (33). Monocytes are recruited to the site of inflammation to differentiate into macrophages, and monocytes/macrophages are an important component of AE (34). Activated monocytes can release a variety of chemokines and cytokines to alter BBB permeability. Macrophages participate in the formation of antigen-presenting cells, which play an important role in antigen processing and presentation, and participate in the activation of T-lymphocyte cells and B-lymphocyte cells to initiate adaptive immunity (31). T cells and B cells are important members of adaptive immunity and play an important role in the progression of AE. Depending on the function, T cells can be divided into CD4^+^ and CD8^+^T cells. CD8^+^T cells play an important role in the body’s immune response to pathogens and tumor surveillance. CD4^+^T cells are helper T cells that play a regulatory role in AE by promoting the differentiation of B cells into plasma cells to promote the generation of antibodies related to AE (35). NLR and MLR originating from blood routine are convenient and inexpensive inflammatory markers, reflecting both innate and adaptive immunity, and changes in NLR and MLR can better reflect the severity of AE. Compared with single leukocyte subtypes such as neutrophils, lymphocytes, and monocytes, NLR and MLR are less affected by factors such as age, gender, and dehydration and can more accurately assess the degree of inflammation.

Currently, the study on NLR and MLR in AE is still in its infancy. A study of AE with small sample size (n = 34) found that NLR was significantly higher in patients than healthy controls, while patients with high levels of NLR tended to be more severe (mRS) at admission compared with AE patients with low levels of NLR (7). A study including 121 cases of NMDAR encephalitis showed that the NLR of severe patients was significantly higher than that of mild patients, and high levels of NLR were an independent risk factor for severe patients (10). Two studies on the treatment effect of AE indicated that high levels of NLR at admission were associated with first-line treatment failure, defined as an improvement in mRS score of less than 1 point after four weeks of treatment (9, 36). Qiu et al. (8) found that high levels of NLR at admission were associated with poor prognosis of AE (mRS > 1), with a median follow-up time of 11 months. Broadley et al. (9) found that the NLR and MLR at admission was not related to the prognosis of AE (mRS ≤ 2 at 12 months and final). However, the mRS was used as the evaluation standard in the above studies, which has great limitations in the assessment of non-motor symptoms in patients with AE, while the sample size is small. In addition, previous studies have shown that MLR is associated with the severity and activity of a variety of immune-related diseases. Huang et al. (37) found that MLR was significantly increased in patients with ankylosing spondylitis compared with patients with nonradiographic axial spondyloarthritis (early stage), and closely related to spine activity, while MLR could be used to evaluate disease severity in axial spondyloarthritis. Hemond et al. (15) showed that MLR was closely related to neurological disability scores and the whole brain atrophy in multiple sclerosis. Suszek et al. (18) found that MLR was associated with systemic lupus erythematosus-dependent organ damage such as cutaneous, mucosal, and kidney, while it was a marker of disease activity. Similar phenomena were also observed in AE, both of which are immune-related diseases. This study found, for the first time, that NLR and MLR were positively correlated with the severity of AE, which was assessed by the CASE and mRS. Subtype analysis showed the same results, and elevated NLR and MLR were independent risk factors for AE disease severity. In addition, the NLR and MLR were not found to be related to the prognosis of AE (mRS ≤ 2 at 12 months). The NLR and MLR were significantly decreased after immunotherapy in AE patients, but the reduction in NLR and MLR after immunotherapy was not found to be associated with prognosis of AE patients.

Since the CASE scale was published in 2019, it has been validated in several studies. Zhang et al. (13) found a positive correlation between the CASE score and the mRS score in 176 patients with AE, with a correlation coefficient of 0.85, while the CASE could predict the functional status at 1 year after discharge. Cai et al. (12) found that the correlation coefficient between the CASE score and the mRS score in 143 AE patients was 0.8. Subtype analysis showed that the correlation coefficients for NMDAR encephalitis (n = 96), LGI1 encephalitis (n = 26) and GABA_B_R encephalitis (n = 17) were 0.84, 0.64 and 0.74, respectively. The correlation coefficient between the CASE score and the mRS score of AE patients in this study was 0.849, and subtype analysis showed that the correlation coefficients of NMDAR encephalitis, LGI1 encephalitis and GABA_B_R encephalitis were 0.868, 0.741 and 0.778, respectively. In this study, the CASE score at admission and age were associated with the poor prognosis of AE patients. Among the three subtypes, multivariate logistic regression analysis showed that the CASE score at admission was not associated with the prognosis of AE patients, partially explained by the small sample size or confounding effects of other factors. Moreover, the correlation coefficients of NLR and MLR with the CASE score were greater than that of NLR and MLR with the mRS score except for NLR and the CASE score in GABA_B_R encephalitis and MLR and the CASE score in LGI1 encephalitis. The CASE scale has a wider distribution than the mRS scale. The CASE scale was more sensitive than the mRS scale in assessing the severity of AE and can distinguish the severity of patients within the same mRS score.

Currently, antibody titers are commonly used to assess the severity of AE in clinical practice. However, its effectiveness is controversial. Butler et al. (38) found that persistent memory deficits in voltage-gated potassium channel complex (VGKC) encephalitis may be associated with high titers of antibodies. Gresa-Arribas et al. (39) found that high antibody titers in NMDAR encephalitis was associated with the presence of a teratoma and/or poor prognosis, and the correlation between CSF antibody titers and clinical relapses was better than that of serum antibody titers. However, A surprising finding was that regardless of whether the prognosis of patients was good or not, the antibody titers of CSF and serum had a decrease at the last follow-up. Arino et al. (40) found that the serum antibodies of 4 patients with LGI1 encephalitis were consistently positive during follow-up, 3 of whom had fully recovered. Shao et al. (41) found that the extent of signal abnormalities in the lesion area in patients with LGI1 encephalitis was positively correlated with the severity of disease (mRS), but not with antibody titers. Moreover, the detection of antibody titers is expensive and time-consuming. In contrast, NLR and MLR derived from blood routine are convenient, inexpensive, and rapid evaluation indicators.

This study contains several limitations. First, this study was retrospective and only completed in a single center, with an inevitable risk of bias. Second, the data of AE antibody titers in serum and cerebrospinal fluid in this study were insufficient to verify the correlation of NLR and MLR with AE antibody titers. Third, the items of the CASE scale are complex, and the accurate CASE score at 12 months for patients cannot be obtained by telephone follow-up. In addition, given that other autoimmune diseases and infections can affect NLR and MLR, we excluded patients with concomitant disease, so the results may not be extrapolated to all patients with AE. A prospective, large-sample, multicenter studies is needed to confirm our conclusion in this study in the future.

In summary, our study is the first to investigate the relationship between MLR and the severity of AE. NLR and MLR, readily available and widespread inflammatory markers, were positively correlated with the CASE score and the mRS score, that is, with the severity of AE. These findings are helpful for clinicians to monitor disease progression and identify potentially severe patients early to optimize clinical treatment decisions. Both the CASE and the mRS can be used to evaluate the severity of AE, with the CASE had greater sensitivity over the mRS and it could be used to predict the prognosis of patients. In addition, the NLR and MLR at admission and whether they decreased after immunotherapy were not associated with the prognosis of AE patients.

## Data Availability Statement

The raw data supporting the conclusions of this article will be made available by the authors, without undue reservation.

## Ethics Statement

The studies involving human participants were reviewed and approved by the ethics committee of the First Affiliated Hospital of Zhengzhou University. Written informed consent to participate in this study was provided by the participants’ legal guardian/next of kin.

## Author Contributions

XC: conceived and designed the study. ZL and YL: collection of data. YW: statistical analysis and interpretation. ZL: wrote the paper. All authors have read and approved the final manuscript.

## Funding

This work was supported by the National Natural Science Foundation of China (81701295).

## Conflict of Interest

The authors declare that the research was conducted in the absence of any commercial or financial relationships that could be construed as a potential conflict of interest.

## Publisher’s Note

All claims expressed in this article are solely those of the authors and do not necessarily represent those of their affiliated organizations, or those of the publisher, the editors and the reviewers. Any product that may be evaluated in this article, or claim that may be made by its manufacturer, is not guaranteed or endorsed by the publisher.
